# Linking Peroxiredoxin and Vacuolar-ATPase Functions in Calorie Restriction-Mediated Life Span Extension

**DOI:** 10.1155/2014/913071

**Published:** 2014-02-03

**Authors:** Mikael Molin, Ayse Banu Demir

**Affiliations:** ^1^Department of Chemistry and Molecular Biology, University of Gothenburg, Medicinaregatan 9C, 413 90 Gothenburg, Sweden; ^2^Department of Molecular Biology and Genetics, Izmir Institute of Technology, 35430 Urla, Izmir, Turkey; ^3^Department of Oncology, Institute of Oncology, Dokuz Eylul University, 35340 Inciralti, Izmir, Turkey

## Abstract

Calorie restriction (CR) is an intervention extending the life spans of many organisms. The mechanisms underlying CR-dependent retardation of aging are still poorly understood. Despite mechanisms involving conserved nutrient signaling pathways proposed, few target processes that can account for CR-mediated longevity have so far been identified. Recently, both peroxiredoxins and vacuolar-ATPases were reported to control CR-mediated retardation of aging downstream of conserved nutrient signaling pathways. In this review, we focus on peroxiredoxin-mediated stress-defence and vacuolar-ATPase regulated acidification and pinpoint common denominators between the two mechanisms proposed for how CR extends life span. Both the activities of peroxiredoxins and vacuolar-ATPases are stimulated upon CR through reduced activities in conserved nutrient signaling pathways and both seem to stimulate cellular resistance to peroxide-stress. However, whereas vacuolar-ATPases have recently been suggested to control both Ras-cAMP-PKA- and TORC1-mediated nutrient signaling, neither the physiological benefits of a proposed role for peroxiredoxins in H_2_O_2_-signaling nor downstream targets regulated are known. Both peroxiredoxins and vacuolar-ATPases do, however, impinge on mitochondrial iron-metabolism and further characterization of their impact on iron homeostasis and peroxide-resistance might therefore increase our understanding of the beneficial effects of CR on aging and age-related diseases.

## 1. Introduction 

Caloric restriction (CR; or dietary restriction [DR]) is the only known intervention that extends the life span of organisms as divergent as yeast, worms, flies, fish, and primates [1, 2], an observation which might indicate the existence of a universal, conserved mechanism of aging. Despite almost 80 years of research since McCay's initial discovery that caloric restriction without malnutrition extended the life span of rats [3], the mechanisms underlying its retardation of the rate of aging are still incompletely understood [4–7]. Since decreased nutrient intake also lowers the incidence of many age-related maladies such as diabetes, cancer, and cardiovascular diseases in several organisms [8], intense efforts at identifying the molecular processes underlying these beneficial effects are underway in the aging researcher community.

Decreased signaling through nutrient-sensing pathways, for example, protein kinase A (PKA), target-of-rapamycin (TOR), or insulin-like growth factor (IGF) pathways [2, 9], is in several model organisms required for life span extension upon CR. These pathways regulate many downstream target processes important for cell growth and stress resistance [10]. However, the many targets and their highly interconnected nature have prevented the identification of targets important for aging. The views of some researchers in the field were, at least until rather recently, that life span extension by CR depends on the combined activity of many gene products acting through multiple pathways [2].

**Figure 1:**
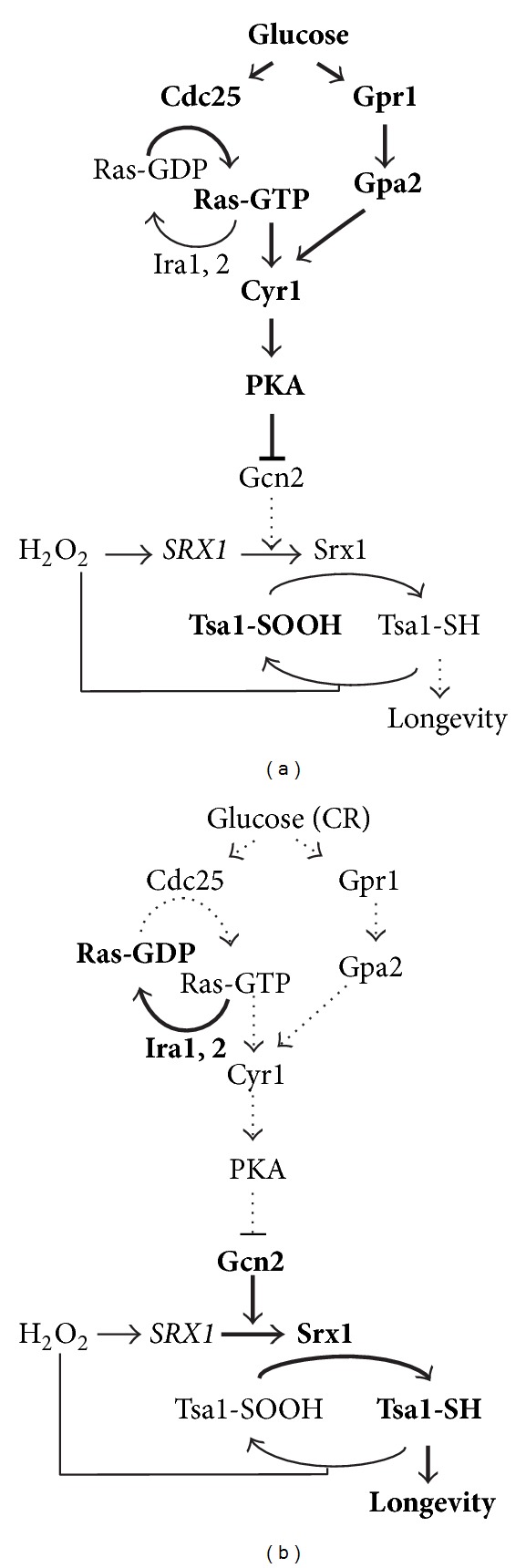
Model for how CR elicits Tsa1 and Srx1-dependent H_2_O_2_ resistance and life span extension. (a) At a high concentration of glucose, when increased signaling through both the Ras-Cyr1 and the Gpr1-Gpa2-Cyr1 signaling branches stimulate PKA activity (Box 1), H_2_O_2_ stress activates Yap1/Skn7-dependent transcription of the *SRX1* mRNA but its translation is attenuated by PKA. As a consequence, Srx1 production is diminished and Tsa1 hyper-oxidized and inactivated. (b) During CR, PKA activity is reduced relieving the translational inhibition of the *SRX1* mRNA in a Gcn2-dependent manner to provide more Srx1 protein and, as a consequence, more reduced, peroxidase-active Tsa1.

In contrast to this hypothesis, two recent reports have pointed to two unique target mechanisms for how CR postpones replicative aging in yeast by counteracting detrimental processes acting at the genesis of aging [11, 12]. We demonstrated that CR, through reduced PKA signaling, activates the peroxiredoxin (Prx) Tsa1, an antioxidant protein reducing H_2_O_2_, to prolong yeast life span [12]. Strikingly, whereas wild-type cells responded to CR (and reduced PKA activity) by an increased life span, *TSA1* mutants did not, identifying Tsa1 as a key enzyme extending life span during CR. In line with this, CR stimulated Tsa1 activity through increasing the levels of the Prx reducing enzyme Srx1, which reduces hyperoxidized (sulfinylated) Tsa1, and ectopically increasing Srx1 levels was sufficient to retard aging in calorie-replete medium (Figure 1). Interestingly, the CR-induced increase in Srx1 levels did not involve increased transcript levels, but rather appeared to result from Gcn2-dependent increased translation of the *SRX1* mRNA (Figure 1(b)). Increased Srx1 levels are expected to increase both the recycling of hyperoxidized Tsa1 and as a consequence also the ability of Tsa1 to reduce peroxide (peroxidase activity). However, Prx are not only H_2_O_2_-reducing enzymes but also function in H_2_O_2_-signaling and in proteostasis (see below) and it is currently not clear which facet(s) of peroxiredoxin function that is/are required for CR-induced longevity.

**Figure 2:**
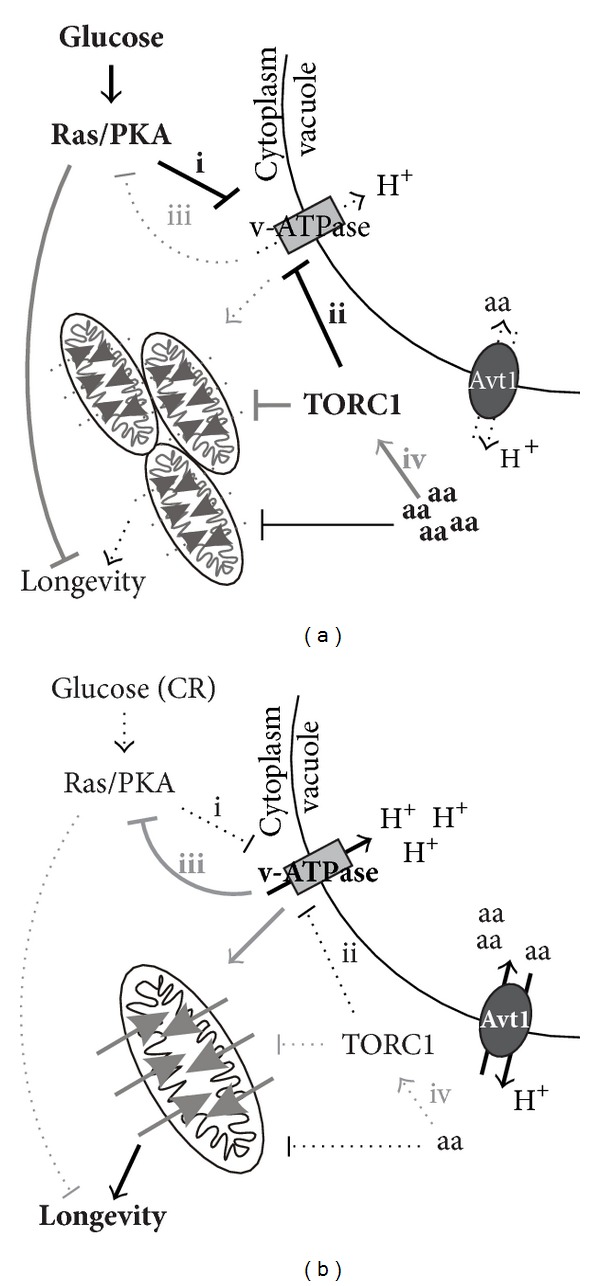
Model for how CR postpones mitochondrial deficiency and aging via stimulating the function of vacuolar ATPase (v-ATPase) and the import of neutral amino acids into the vacuolar lumen. (a) Upon high glucose levels increased Ras-cAMP-PKA (Ras/PKA) activity inhibits v-ATPase function (arrow i) and thereby also the neutral aminoacid transporter Avt1 leading to the accumulation of neutral amino acids in the cytoplasm. Among these, leucine has been proposed to activate TORC1 through the leucyl-tRNA synthetase LeuRS and the Gtr1 GTPase (arrow iv). Cytosolic amino acid accumulation is thought to decrease mitochondrial function and to stimulate aging, possibly via increased TORC1 signaling. Increased TORC1 activity would also be expected to further inhibit v-ATPase function (arrow ii). Reduced v-ATPase function might also be expected to releive v-ATPase inhibition of Ras-PKA via cytosolic alkalinization (arrow iii). (b) CR and reduced glucose inhibition of v-ATPase function by Ras-PKA (arrow i) stimulates Avt1-mediated uptake of neutral amino acids. Lower cytoplasmic leucine levels would be expected to reduce TORC1 activity (arrow iv) and TORC1-mediated repression of v-ATPase activity (arrow ii). Similarly, increased v-ATPase function might be hypothesized to increase the inhibition of Ras-PKA activity, possibly via cytosolic acidification. Mitochondrial functions are maintained under conditions of reduced Ras/PKA and TORC1 activity as well as reduced levels cytosolic amino acids, which stimulates longevity. Arrows in black represent mechanisms at least in part experimentally verified to be in operation in aging cells [11] whereas arrows in grey indicate mechanisms inferred based on recent data implicating cytosolic pH and v-ATPase in the regulation of Ras-PKA [14, 16] as well as cytoplasmic leucine levels in the regulation of TORC1 signaling [13].

Interestingly, a recent report from the Gottschling lab identified increased vacuolar pH as an early-age promoter of age-induced mitochondrial depolarization and fragmentation leading to replicative aging in yeast (Figure 2(a), [11]). Caloric restriction, as well as reducing the activities of conserved nutrient signaling pathways (e.g., Ras-cAMP-PKA and TORC1), delayed an age-induced loss of vacuolar acidity suggesting that the control of vacuolar pH also constitutes a target process regulated by CR (Figure 2, arrows i and ii, [11]). Mutating subunits of the vacuolar proton-translocating ATPase (v-ATPase) leads to increased vacuolar pH and accelerated aging (Figure 2(a)). Conversely, restoring vacuolar acidity in aging cells retarded aging (Figure 2(b)), suggesting that the control of vacuolar pH critically regulates aging. The authors furthermore observed that increasing vacuolar pH decreased the import of cytosolic amino acids into the vacuole through the H^+^-neutral amino acid antiporter Avt1, and both mitochondrial dysfunction and replicative aging were in part coupled to this reduced transport (Figure 2(a)). How increased levels of cytosolic amino acids induce mitochondrial dysfunction and replicative aging was not addressed. As we will discuss later, however, both v-ATPase and a cytosolic neutral amino acid (leucine) have been implicated in the regulation of nutrient signaling [13–15], raising the question whether v-ATPase and cytosolic amino acids affect aging via feedback regulation of nutrient signaling pathways.

The purpose of this paper is to review the physiological impact of both Prx-mediated stress defense and vacuolar pH control and to pinpoint possible common denominators between these two apparently distinct target mechanisms proposed for CR-mediated life span extension. We will then point to outstanding questions that need to be resolved to further understand the beneficial impact of caloric restriction on aging and age-related diseases. To achieve this, we will first review the roles of ROS and peroxiredoxins in aging and in CR-mediated longevity. Next, we will shortly consider nutrient and glucose signaling in general and through the lenses of the free radical theory of aging in particular, since these signaling pathways are conserved mediators of CR life span extension in most organisms. Following this, we will discuss the cellular roles of v-ATPases including recent observations that these enzymes, via regulating intracellular pH homeostasis, are implicated in the regulation of nutrient signaling. On a final note, we will discuss points of intersection between v-ATPases and Prxs identified here, which include recent papers implicating both pathways in iron metabolism.

## 2. Reactive Oxygen Species (ROS) in Aging and in Caloric Restriction-Mediated Life Span Extension: Damaging Agents or Signal Transducers?

In aerobic organisms, incomplete reduction of oxygen in the mitochondrial respiratory chain leads to the production of reactive molecules called reactive oxygen species (ROS) [17]. These reactive molecules mainly include hydrogen peroxide (H_2_O_2_), superoxide anion (O_2_^−^), and hydroxyl radical (OH^·^) and they may damage macromolecules, including proteins, lipids, and nucleic acids [18].

The most widely accepted aging theory, the free radical theory, proposed by Denham Harman in 1956, states that damage caused on biomolecules by ROS/free radicals might be an underlying cause of the aging process [19]. Consistent with this hypothesis, H_2_O_2_ levels have been shown to increase with the replicative age of yeast mother cells [20, 21] and with the chronological age of both rats and mice [22, 23]. Whereas H_2_O_2_ in itself mainly oxidizes cysteine or methionine residues, its conversion into the highly reactive OH^·^ via metal-catalyzed oxidation reactions, may inflict widespread damage [18]. Macromolecular damage has been observed to increase with age in many organisms [18, 24–26]. Furthermore, a correlation between the amount of oxidized proteins and the rate of aging was reported in flies and in cultured human fibroblasts [18, 27]. The levels of oxidized proteins have also been shown to increase with age in rat liver and brain extracts. In many organisms studied, CR post-pones the increased production of ROS seen with age [28, 29], increases the resistance to oxidative insults [12, 30, 31], and reduces oxidative damage [32, 33].

On the other hand, observations that too low levels of ROS lead to deficiencies in proliferation and immune responses [34], suggest that a balanced level of oxidants is necessary for the normal functions of cells and organisms. Illustrating this, proliferation and growth factor signaling in multicellular organisms involve controlled production of O_2_^−^ or H_2_O_2_ by, for example, NADPH oxidase enzymes [35]. Superoxide anion is instable due to its reactivity and it cannot diffuse through membranes, which makes it a poor signaling molecule [17, 36]. In contrast, the half-life of H_2_O_2_ is longer and since it is an uncharged molecule it can diffuse through membranes [37]. H_2_O_2_ can also be transported across biological membranes through aquaporin channels. Therefore, it is not surprising that most ROS signaling involves signal transmission via H_2_O_2_ [17].

ROS signaling has been implicated in a variety of cellular events, for example, development and growth, oxidative stress resistance and even in the retardation of aging [38]. In particular, adaptive responses to moderately increased ROS levels have been proposed to induce antioxidant defences and hence resistance to ROS and longevity upon CR [38, 39]. In a recent study, thiol-peroxidases were shown to be the main receptors for H_2_O_2_ signaling in yeast cells, since the absence of all the eight thioredoxin-dependent peroxidases (five Prxs and 3 glutathione-peroxidase homologs) resulted in an inability to sense and respond to externally added H_2_O_2_ [40]. However, for most of the thiol peroxidases/Prxs, the signal transduction pathways regulated are not known. A dual role in peroxide scavenging and signaling has been proposed for thiol peroxidases in several organisms [41–43].

Stimulation of longevity by ROS signaling is suggested by the observation that treatment of *C. elegans* worms with low levels of the superoxide anion generator paraquat increased lifespan by more than 50% [44]. Such a prolongevity role of ROS is, however, only seen at moderately increased ROS levels, since higher doses of paraquat reduced life span and low levels of the drug were toxic in worms lacking detectable SOD activity. Growing yeast cells under CR (0.5% glucose or lower) appears to lead to both higher respiration [45] and higher mitochondrial ROS production, as suggests the life span of cells lacking the mitochondrial SOD, *SOD2*, that is normal at high glucose levels but drops precipitously at glucose levels of 0.5% or lower [46]. This deficiency could be suppressed by the addition of the antioxidant ascorbic acid supporting the idea that *sod2* mutants suffer excessive ROS levels at reduced glucose levels. Similarly, CR has been observed to increase respiration and mitochondrial ROS production in mice and worms and these increases were reported to be required for the induction of both antioxidant defences and longevity [47, 48].

In summary, ROS appear to extend life-span, presumably by inducing adaptive ROS signaling, at low levels, but are toxic at higher levels. CR may mitigate oxidative damage by stimulating the cellular ROS defence capability through an increase in mitochondrial ROS production.

## 3. Peroxiredoxins in ROS Defence and in the Retardation of Aging

Prxs are an evolutionarily highly conserved antioxidant enzyme family which members reduce intracellular peroxide levels through a cysteine-based mechanism [40, 49]. Prxs were first discovered in yeast [50], *E. coli* and *S. typhimurium* [50, 51] but are now found to be widespread across phylogeny with one or more members typically being abundantly expressed in many aerobic organisms. Prxs are divided into groups based on the number and the position(s) of catalytic cysteine residues. The major subclass is the 2-Cys Prxs that form homodimers and catalyze the reduction of peroxide via two conserved catalytic cysteine residues [52, 53]. Catalysis is initiated by the reduction of H_2_O_2_ by a conserved N-terminal cysteine residue (the peroxidatic cysteine) which, in turn, oxidizes to the sulfenic acid form (Cys-SOH). In 2-Cys Prxs, the peroxidatic Cys-SOH then condenses with the second catalytic cysteine residue present in the other monomer to form a disulfide that is reduced by thioredoxin, thus completing the catalytic cycle. However, the peroxidatic Cys-SOH might react again with H_2_O_2_, when the levels of it are high, which leads to the formation of a sulfinic acid (Cys-SOOH), and enzyme inactivation (Figure 1). Such inactive form of Prx is slowly reactivated by ATP-dependent reduction of the sulfinic acid by sulfiredoxin (Srx1, [54]).

Prx deficiency accelerates aging in yeast, worms, flies, and rodents [42, 55–57]. In addition, increasing the levels of a neuronal Prx in flies [55] or increasing sulfiredoxin levels in yeast [12], extends life span (Figure 1(b)), suggesting that the anti-aging function of Prxs is conserved [58]. In addition, mice deficient in PrxI age prematurely and develop several malignant cancers indicating that Prxs prevent a prevalent group of age-related diseases [56]. Yeast cells lacking Tsa1 suffer from increased mutation rates and genome instability [59], which at least to some extent has been linked to increased oxidative DNA damage [58, 60].

The genome of the budding yeast *S.cerevisiae* encodes for eight thioredoxin-dependent peroxidases. Five of these are Prxs proper (Tsa1, Tsa2, Ahp1, Dot5, and Prx1) and the other three Prx-like enzymes with sequence homology to glutathione peroxidases (Gpx1, Gpx2, and Gpx3) [40, 61, 62]. Although these Prxs are thought to constitute an important part of the antioxidant arsenal, *S. cerevisiae* cells lacking the eight yeast thioredoxin-dependent peroxidases are viable, do not appear to contain increased levels of ROS and can withstand a certain level of oxidative stress [40]. Interestingly, this mutant still suffers from a reduced replicative lifespan which might therefore be caused by the lack of functions other than direct ROS detoxification. However, these data somewhat contradict earlier reports that yeast cells lacking all five Prxs contain increased ROS levels and are genomically unstable [63]. To settle this issue, specifically in the context of aging, it will be important to monitor intracellular ROS levels using modern, more sensitive and specific ROS sensors [64, 65] in aging Prx-sufficient and -deficient cells [20, 21].

Tsa1 contributes 91% of all Trx dependent peroxidase activity in yeast cells [62]. Yeast Tsa1 and mammalian Prx1 share 65% amino acid identity [66]. Similar to Tsa1, the 2-Cys Prxs Prx1, and Prx2 are also very abundant in mammalian cells, which despite poor catalytic efficiency might indicate that they are important in H_2_O_2_ scavenging [67].

Tsa2 is a 2-Cys Prx 86% sequence identical to Tsa1 but that is expressed only at ~80-fold lower levels than Tsa1 [68]. In agreement with its low expression, cells lacking Tsa2 are not deficient in ROS defence, but in fact, paradoxically, appear to grow better than wild-type cells in the presence of H_2_O_2_ [12]. However, in cells lacking *TSA1,* the levels of Tsa2 are increased [69] and cells lacking both *TSA1* and *TSA2* are more sensitive to H_2_O_2_ than the *TSA1* single mutant [70], indicating that the two cytosolic Prxs cooperate in peroxide defence.

2-Cys Prxs are multifunctional enzymes which, in addition to their peroxidase activity required to cope with oxidative stress, can act as chaperone holdases to counteract protein damage and as H_2_O_2_ signaling devices [71, 72]. During peroxidase catalysis, further oxidation of the peroxidatic cysteine of a 2-Cys-Prx from sulfenic acid into sulfinic acid leads to the inactivation of its peroxidase activity. Interestingly, hyperoxidation stabilizes the formation of high-molecular-weight Prx complexes (dodecameric and higher order decameric derivative forms) [73, 74], which carry increased chaperone activity [75]. Similar alterations in quaternary structure were observed upon increased temperature or reduced pH and were also found to stimulate the ability of Prxs to prevent the aggregation of model proteins *in vitro* [74, 76]. Reversion of the sulfinylated form of Tsa1 by ATP- and sulfiredoxin-dependent reduction indicates that sulfinylation is a redox-switch that alters the function of Prxs from a peroxidase to a chaperone. Prxs have been shown to interact with a multitude of signaling proteins and the redox-dependent oligomerization of 2-Cys-Prxs may therefore be important also in the regulation of signaling [75].

Interestingly, accumulation of the hyperoxidized forms of the yeast and rat Prxs, Tsa1, and PrxIII, have been reported upon aging [12, 77], indicating that Prx inactivation may be a common phenotype in aging organisms (Figure 1(a)). It is, however, not clear whether increased hyperoxidation with age is due to increased levels of H_2_O_2_ [20–23] or deficient Srx1-mediated Prx de-sulfinylation [12] and whether this in turn controls a switch to the chaperone-function [76] or modulates Prx-dependent H_2_O_2_ signaling [40, 78].

In summary, Prxs are multifunctional enzymes that retard aging and it is currently unknown which of their functions is/are important to retard aging. It appears that the signaling role of Tsa1 may be discarded, however, since cells lacking *TSA1* do not appear to display altered gene expression [12, 40] yet age at a faster rate and fail to extend life-span upon CR [12]. The function of yeast Tsa1 in CR-mediated longevity appears to require Srx1 since the recycling of hyperoxidized Tsa1 is necessary and sufficient for CR life span extension. However, it is currently not clear what is the beneficial effect of Tsa1 recycling during aging; the consequential increased Tsa1 peroxidase activity or a function linked to enzyme chaperone activity? Given the unique ability of Prx peroxidase activity to scavenge low levels of endogenous peroxide to protect the genome [67], the determination of mutation rates and/or DNA damage in aging CR cells would give an indication of the importance of the scavenging function during aging.

## 4. Calorie Restriction, Glucose Signaling, and the Link to the Free Radical Theory of Aging

The three conserved nutrient sensing kinases that mediate life span extension by CR in yeast are the protein kinase A (PKA), the target-of-rapamycin-complex 1 (TORC1) kinase, and the Akt/protein kinase B/ribosomal S6 kinase homologue Sch9 [4, 79, 80]. Partly reduced activites in any of these three pathways mimic the effect of calorie restriction on aging. Accordingly, partial inactivation of PKA catalytic subunits, cAMP synthesis, or regulatory proteins of the pathway (e.g., Cdc35/Cyr1, Cdc25, Gpr1, and Gpa2 in yeast) are frequently used as calorie restriction mimetics and mitigate aging and/or age-related diseases in mice [9, 81, 82], *Drosophila* [83], and yeast ([4, 84], Figure 1(b)). Since both the TORC1/Sch9 and Ras-cAMP-PKA pathways control mitochondrial activity and stress-defenses, nutrient signaling clearly impinges on pathways relevant in the context of the free radical theory of aging [10]. For example, in both yeast cells and mouse adipose tissue TORC1 negatively controls respiration [10, 85]. Similarly, the reprogramming of yeast metabolism upon glucose depletion and growth on respiratory carbon sources requires reduced activity of the Ras-cAMP-PKA pathway, which inhibits of mitochondrial biogenesis and mitochondrial activity [10]. Many yeast antioxidant enzymes are also under negative control of the Ras-cAMP-PKA pathway, for example, cytosolic catalase [86], all five yeast Prxs [12, 87–89] and the mitochondrial SOD [90]. TORC1 similarly represses at least some of the yeast antioxidants. Most of the studies on CR-mediated longevity in yeast have focused on the impact of reduced glucose levels on aging and a short review of yeast glucose signaling can therefore be found below (Box 1).

In conclusion, the major regulatory impact of the nutrient signaling pathways on mitochondrial activity and on antioxidant defence pathways indicate that the mechanisms underlying their effects on the rate of aging and CR-induced longevity is compatible with the free radical theory of aging. In agreement with this, decreased nutrient signaling increases the resistance to oxidative stress, presumably at least in part because increased ROS defences more than offset an increased mitochondrial activity.

**Box 1 (*yeast glucose signaling*)**

Glucose is the most abundant monosaccharide in nature and a rich carbon source that is preferred as a primary energy source by a variety of organisms, from yeast to humans. In *S. cerevisiae*, it has been shown that upon glucose addition the expression of about 40% of the genes in the genome is altered, indicating a substantial reprogramming of gene expression [91]. The response to glucose is mainly regulated by the Ras-cAMP-PKA pathway [92]. Yeast has a G protein coupled receptor (GPCR) (Gpr1) that can accommodate glucose or sucrose as ligands to activate the G-protein Gpa2 [93]. Gpa2 in turn stimulates the PKA signaling pathway via adenylate cyclase (Cyr1) and cAMP levels (Figure 1, [93]). However, the most important part of the response to glucose is thought to involve the stimulation of Ras activity (Figure 1, [94, 95]), the mechanism of which still remains elusive despite extensive research. Ras2 (and to a minor extent Gpa2) activation more or less fully recapitulates the transcriptional response to glucose addition through stimulating PKA activity [91], arguing that PKA activation is sufficient for the response to glucose. Whereas glucose still stimulates cAMP synthesis in the absence of Gpr1 or Gpa2, the cAMP increase seen upon glucose addition is lost in cells deficient in Ras [96]. Of note, glucose phosphorylation is required for Ras activation and cAMP synthesis indicating that the Ras proteins may respond to the levels of a glucose metabolite.

## 5. Roles for Intracellular pH Homeostasis and Vacuolar ATPases in Nutrient Signaling

Recent data on yeast nutrient sensing suggest that nutrient signaling is intimately connected to v-ATPase function and intracellular pH homeostasis. V-ATPase deficiencies in aging cells thus likely directly impinge on the activation of nutrient signaling pathways. To better understand this connection, we will next shortly review the cellular roles of v-ATPases.

H^+^-ATPases are important contributors to cellular pH homeostasis. In addition to being localized at the vacuolar membrane, where they (v-ATPases) acidify the vacuole, they are also found at the plasma membrane of several mammalian cell types [97–100], where they pump protons towards extracellular space [101]. In yeast cells, pH homeostasis is maintained by two proton pumps; Pma1, which resides at the plasma membrane, and v-ATPases, which reside within the membranes of multiple organelles (in particular the vacuole and the Golgi). These two H^+^-ATPase pump systems seem to function coordinately since any defect in the v-ATPase causes altered localization of the plasma membrane H^+^-ATPase, Pma1, presumably to maintain intracellular pH homeostasis [102].

All eukaryotic v-ATPases consist of two subcomplexes, V_0_, and V_1_, that are formed from 6 and 8 subunits, respectively [103]. Deletion of any v-ATPase subunit is lethal in all organisms except fungi [104]. In yeast cells, v-ATPase subunit deletions lead to a growth phenotype called the “*vma* phenotype,” which is characterized by an inability to grow on respiratory carbon sources, a sensitivity to increased extracellular pH, to heavy metals and to oxidants [104]. Accordingly, several genes encoding vacuolar proteins, for example, those important for vacuolar acidification, were identified in a genome-wide screen for genes important for oxidant tolerance suggesting that v-ATPase function is required for proper antioxidant defences [105].

In yeast, pH homeostasis is intimately linked to glucose utilization. The addition of glucose to starved cells leads to a rapid and very transient cytosolic acidification (30 seconds, [106, 107]), which is thought to be caused by the initiation of glycolysis, followed by its alkalinization through the H^+^-ATPase activities of Pma1 [108] and the v-ATPase [102] upon which growth recommences [106] and cAMP levels increase [14]. Cytosolic alkalinity correlates closely with glycolytic rates [14] and would eventually be expected to inactivate v-ATPase function because of reduced substrate availability (Figure 2(a), [102]). Mutants lacking v-ATPase activity, grown at steady state in high glucose, display acidic cytosolic pH [14, 102], in agreement with a role for v-ATPase in cytosolic alkalinization. Upon glucose withdrawal, the v-ATPase is inactivated by disassembly of its two subunits, which lowers cytosolic pH and reduces the activity of the PKA pathway [14]. Presumably, upon reduced glucose (CR), the v-ATPase is subsequently reactivated to promote vacuolar acidification and/or to maintain cytosolic pH homeostasis (Figure 2(b), [11]). In agreement with this, cytosolic pH drops continuously as yeast cells grow and consume glucose [16], but cytosolic and vacuolar pH have not been measured during replicative aging of CR cells. In addition, although the measurement of vacuolar pH was used to argue for a role for v-ATPase mediated vacuolar acidification in CR-mediated longevity [11], cytosolic pH was never measured and thus the full scope of increased v-ATPase activity in aging CR cells was not assessed. These data thus raise the question whether v-ATPase-mediated regulation of cytosolic pH contributes to CR life span extension by influencing nutrient signaling (Figure 2). Importantly, cytosolic pH appears intimately connected to the cellular nutrient status since both glucose starved cells and cells grown on respiratory carbon sources show reduced cytosolic pH [16]. In fact, cytosolic pH was proposed to control the cell division rate both in yeast cells [16] and in *Xenopus* oocytes [109]. Thus, to better understand life span extension upon CR it would be important to monitor also cytosolic pH as cells age.

The assembly/disassembly of v-ATPases is fast and constitutes an important regulatory mechanism of v-ATPase function. The role of the Ras-cAMP-PKA pathway in these events is, however, controversial. As mentioned above, v-ATPase assembly is stimulated by glucose *in vivo *[110] and this process was reported independent on cAMP-PKA and the conventional glucose signaling pathways, but dependent on glucose metabolism beyond glucose-6-phosphate [111]. V-ATPase complex integrity independent on the Ras-cAMP-PKA pathway is supported by the unaltered disassembly of the Vma5 subunit of the V_1_ domain upon glucose withdrawal in *ira2* mutant cells, as observed by microscopical observation of a Vma5-RFP fusion protein [14]. In contrast, a genetic screen in yeast for mutants lacking v-ATPase disassembly upon glucose withdrawal identified loss of *IRA1*, *IRA2* and *BCY1* gene functions [101], which negatively regulate Ras-cAMP-PKA (Figure 1, Table 1). Furthermore, vacuolar acidity seems reduced in *ira2* mutant cells *in vivo* [11], also suggesting that *IRA2* is essential for proper v-ATPase function (Figure 2(a), Table 1). Thus it is not clear at this point if v-ATPase is acting upstream of or downstream of the Ras-cAMP-PKA pathway or if it participates in a complex feedback loop involving Ras-cAMP-PKA (Figure 2). Reciprocal regulation of PKA and v-ATPases has been proposed to reinforce PKA activation and v-ATPase assembly upon glucose sensing [14] but the roles of glucose and the Ras-cAMP-PKA pathway in v-ATPase assembly and disassembly clearly need further clarification.

As discussed earlier, part of the increased longevity and the maintenance of mitochondrial function achieved by vacuolar acidification could be linked to the uptake of neutral amino acids into the vacuole (Figure 2(b), [11]). Supporting this, overproduction of the vacuolar neutral amino acid antiporter Avt1 reduced age-induced mitochondrial dysfunction and extended life span. In addition, overexpression of the v-ATPase subunit Vma1 only partly suppressed age-induced mitochondrial dysfunction in cells lacking *AVT1* (Figure 2(b), [11]). In this respect it is interesting that v-ATPase and the proton-driven transport of lysosomal amino acids have been implicated in the activation of mammalian TORC1 in response to amino acids [15]. In yeast, TORC1 is activated by cytosolic leucine, the most frequently utilized (and neutral) amino acid (Figure 2(b), arrow iv), by a mechanism involving the cytoplasmic leucyl-tRNA synthetase LeuRS charged by leucine and the GTPase Gtr1 [13]. It is thus conceivable that the reduced v-ATPase-function and vacuolar proton-gradient in aging cells promotes both PKA and TORC1 activation via inefficient transport of neutral amino acids into the vacuole.

In summary, CR, via reduced glucose intake, may repress PKA activity through increased v-ATPase function and reduced cytosolic pH (Figure 2(b), arrow iii). Accordingly, maintained v-ATPase activity would extend life span via feedback regulation of both Ras-PKA- and TORC1 pathways, the latter via reducing cytosolic leucine levels. Similarly, amino acid-restriction could be expected to efficiently reduce TORC1 activity through increased v-ATPase function and Avt1 controlled amino acid transport into the vacuole (Figure 2(b), arrows ii & iv), thus also repressing Ras-cAMP-PKA activity (Figure 2(b), arrow iii). Future studies should address the roles of v-ATPase in feedback regulation of nutrient signaling pathways and in the cross-talk between the pathways.

## 6. A Link between Calorie Restriction, Vacuolar Acidification, and Peroxiredoxins in the Regulation of Aging

The compilation of data from different organisms identified vacuolar functions among several conserved genes essential for CR to extend life span [118]. In addition, a recent study examining the life spans of 166 yeast deletion mutants upon CR suggested that both loss of vacuolar pH control or antioxidant defences negatively affect life span during CR [46], suggesting that both processes are essential for CR life span extension. It is clear from the studies reviewed here that both Ras-PKA and TORC1 interact reciprocally with v-ATPase functions. Furthermore, the expression and activites of Prxs is regulated by nutrient signaling pathways during CR or changes in carbon source in the media [12, 88].

Regarding potential connections between v-ATPase and Prxs, the pH-regulated oligomerization and chaperone activity of a *Schistosoma mansoni* Prx *in vitro* [74] is certainly intriguing but the occurrence of Prx oligomerisation at acidic pH is currently unknown *in vivo*. As mentioned above, in starved yeast cells pulsed with glucose, cytosolic pH has been reported initially to reach as low as 5.3 [106], which is still significantly higher than the pH where oligomerisation of the *S. mansoni* Prx was observed *in vitro* (pH 4.2) [74]. Thus, it will be interesting to know whether Prxs from other organisms also oligomerize at acidic pH and at *in vivo* relevant values.

Another still intriguing connection between v-ATPase and oxidative stress, and hence the Prxs, is the observation that a yeast mutant lacking v-ATPase function (*vma2*Δ) is exquisitely sensitive to H_2_O_2_ [112] and less affected by other oxidants (Table 1) when grown under conditions that minimize the *vma* phenotype (i.e., acidic extracellular pH). Under these conditions *vma2*Δ cells also display elevated DCDF-DA staining, that might indicate elevated intracellular ROS, and increased protein carbonylation [112]. In addition, *vma2*Δ*tsa1*Δ mutants display a severe synthetic growth phenotype indicating that they share a function important in cell physiology [115].

Iron metabolism may be yet another connection between v-ATPases and Prxs. In cells lacking *VMA2*, microarray analyses revealed prominent induction of genes that are involved in iron metabolism under the control of the transcription factor Aft1 [112]. The link between v-ATPase and iron metabolism was further supported by the synthetic lethality of a strain lacking both *VMA2* and *AFT1* [115]. In yeast, iron is indirectly sensed through the amount of Fe/S-cluster proteins that are matured in the mitochondrial matrix [119, 120], which itself is a function of cellular iron availability and of mitochondrial Fe/S-biogenesis. In a follow-up study, the *vma2*Δ mutant was, in accordance with a defect in mitochondrial Fe/S-cluster biogenesis, shown to contain total iron levels similar to the wild-type but, importantly, to display reduced activity of aconitase, a mitochondrial Fe-S cluster enzyme [115]. Importantly, adding a weak acid to wild-type cells which, like deficient v-ATPase function, is expected to acidify the cytosol, and mitochondria [106] was sufficent to induce Aft1 transcription [115]. These data indicate that v-ATPase-regulated pH homeostasis is crucial for mitochondrial Fe-S cluster biosynthesis/biogenesis. However, Fe-S clusters have been reported to become unstable at low pH [121, 122] indicating that v-ATPase-regulated pH homeostasis also may impinge on Fe/S-cluster stability. Interestingly, deficient mitochondrial Fe-S cluster biogenesis and growth arrest in daughters of replicatively aged yeast mother cells was earlier linked to the loss of mitochondrial DNA and mitochondrial membrane potential [113], suggesting that mitochondrial Fe-S clusters become instable with age as a result of mitochondrial DNA damage. Reciprocal crosstalk between mitochondria and v-ATPase regulated pH homeostasis is suggested by the observation that defects associated with the loss of mitochondrial DNA (e.g., slow growth and defective mitochondrial protein import) can be suppressed by loss of v-ATPase function [114] (Table 1). Taken together, these data raise the possibility that reduced vacuolar acidity in aging cells [11] may be an adaptation to suppress certain phenotypes associated with mitochondrial deficiency, but which on the contrary might exacerbate yet others, such as reduced mitochondrial Fe/S cluster biogenesis. Future studies are clearly necessary to identify mechanisms causing the loss of v-ATPase function with age as well as the intricate interplay between v-ATPase mediated pH homeostasis and mitochondrial function.

Is there now a link between Prxs and any of these phenotypes? Interestingly, *TSA2* transcription, but not the transcription of other antioxidant genes, is increased upon decreased v-ATPase function (in cells treated with concanamycin A or cells lacking *VMA2*, Table 1, [112, 115]). In line with a role in iron metabolism, increased *TSA2* transcription in a *vma2*Δ mutant could be suppressed by supplementing cells with iron. In addition, in cells lacking *TSA2* (*tsa2*Δ and *vma2tsa2*Δ), but not in peroxidase-negative mutants, expression of the Aft1-target gene *FIT2 *is increased (4-fold and 2-fold, resp.), indicating that *TSA2* represses Aft1-activity in a manner independent of Tsa2 catalytic activity. Furthermore, Tsa1 and its peroxidatic cysteine appear necessary to support aerobic growth of cells lacking high affinity iron transport across the mitochondrial membrane by *mrs3mrs4* deficiency [117] (Table 1). Interestingly, Mrs3- and Mrs4-deficient cells require a repressor of the iron regulon, Fra1, for aerobic growth [117] and suppression of the aerobic growth defect of *mrs3mrs4* deficient cells by Fra1 required *TSA1* (Table 1). Taken together, repression of Aft1 transcription by Tsa2 and the interactions of both Tsa1 and Tsa2 with the iron regulatory protein Fra1 indicate a link between Prx and iron metabolism that will need to be elucidated at the molecular level.

Hence, it seems that both vacuolar pH control and Prx activity are linked to iron homeostasis and Fe/S cluster biogenesis and a further characterization of their impact on these vital processes appears important. More specifically, a better understanding of the role of Prxs in longevity requires more careful assessment of their described roles in peroxide scavenging and in signaling in aging cells. Since v-ATPases appear to impinge on mitochondrial Fe/S-cluster stability and both cytosolic Prxs in yeast appear to interact closely with iron metabolism, a better understanding of their roles in these processes might also help to unravel how Prxs and v-ATPases contribute to slow down the rate of aging during caloric restriction.

## Figures and Tables

**Table 1:** Common denominators of v-ATPase and peroxiredoxin functions in yeast which might impinge on calorie-restriction-mediated life span extension. For more details see the text. O/e: overexpression; Fe: iron; Δ: deletion mutant.

	Protein kinase A	H_2_O_2_ resistance	Fe-metabolism
Vacuolar ATPase	v-ATPase disassembly [101] and v-ATPase-driven vacuolar acidification [11] both inhibited in strains lacking *IRA2 *	*vma2*Δ (V_1_ domain) and *vma3*Δ (V_0_) mutants very sensitive to H_2_O_2_ at permissive pH 5 [112]	O/e *VMA1* or *VPH2* suppressed age-induced loss of mitochondrial membrane potential [11]. Age-induced loss of mitochondrial DNA causes loss of membrane potential and defects in Fe/S-cluster biogenesis [113]
	v-ATPase activity regulates PKA activity upon glucose addition [14]	*vph1*Δ (V_0_ vacuole) moderately sensitive to H_2_O_2_ at pH 5 [112]	v-ATPase inhibition by concanamycin A caused rapid loss of mitochondrial membrane potential [11]
			Reduced v-ATPase function suppresses defects associated with the loss of mitochondrial DNA and membrane potential [114]
			Aft1 is required for the survival of a strain lacking *VMA2* [115]

Peroxiredoxins	Tsa1 peroxidase function stimulated at low PKA activity (*gpa2*Δ, *gpr1*Δ, *cdc35-1*, o/e *PDE2*) [12]	*tsa1*Δ sensitive to H_2_O_2_ but not tert-butyl-OOH [116]	O/e *FRA1* suppressed slow growth and increased Aft1-dependent transcription in a strain lacking mitochondrial high-affinity Fe-transport (*mrs3*Δ *mrs4*Δ), in a *TSA1* dependent manner; Tsa1 interacts physically with Fra1 [117]
			Tsa1 and Tsa1Cys48 are required for aerobic growth of an *mrs*3Δ*mrs4*Δ strain [117]
	Tsa2 levels increased at low PKA activity (*ras2*Δ) [88]	*tsa2*Δ slightly resistant to H_2_O_2_ [12], *tsa1*Δ *tsa2* more sensitive than *tsa1*Δ [70]	*Tsa2*, as well as Tsa2C48, represses the expression of an Aft1-target (*FIT2*) in wt and *vma2*Δ, *Tsa2* binds to Fra1 in *vma2*Δ, increased *TSA2* levels in *vma2*Δ suppressed by Fe supplementation [115]
